# Data on the life cycle carbon emission in Kenyan, Rwandan, and Tanzanian grid electricity generation and transmission systems

**DOI:** 10.1016/j.dib.2021.107692

**Published:** 2021-12-08

**Authors:** Enock Chambile, Nelson Ijumba, Burnet Mkandawire, Jean de Dieu Hakizimana

**Affiliations:** a African Centre of Excellence in Energy for Sustainable Development, College of Science and Technology, University of Rwanda, KN 73 St, P.O. Box 3900, Kigali, Rwanda; b Department of Geography and Environmental Studies, Solomon Mahlangu College of Natural and Applied Sciences, Sokoine University of Agriculture, P.O. Box 3038 Chuo Kikuu, Morogoro, Tanzania; c School of Engineering, University of KwaZulu Natal, Howard College Campus, P/Bag X5401, Durban 4041, South Africa; d Faculty of Engineering, Malawi University of Business and Applied Sciences, P/Bag 303, Chichiri, Blantyre 3, Malawi

**Keywords:** Clean technologies, Electrical power system capacity, Carbon emissions data, Life cycle carbon emission estimation parameters

## Abstract

This paper presents data for the estimation of the life cycle carbon emission in Kenyan, Rwandan, and Tanzanian grid electricity generation and transmission systems. Data was collected and estimated using the developed life-cycle carbon emission inventory (LCCEI) algorithm implemented through Excel tabs (LCCEI Excel worksheets). The data acquired through the LCCEI modelled parameters (Chambile et al., 2021). The presented dataset shows the results of the developed data collection model. The activity data were obtained from specialized data sources. Some information was obtained through meetings with relevant institutional actors and experts of national and regional power institutions as well as expert judgement. However, most of the data were also obtained from the reviewed published reputable sources, such as the scientifically indexed conference proceedings and journals. The obtained data are presented in this article and in a Mendeley data repository. The compiled data can also be customised and coded to commonly used evaluation software to enhance its open use by scientists, practitioners, and policymakers at national, regional and global levels.

## Specifications Table

**Table:** 

Subject	Energy
Specific subject area	Energy and Environmental Sciences, Engineering, and Technology
Type of data	Table, text, Excel Spreadsheet and figure.
How data were acquired	The existing primary and secondary data, and developed new case-specific data were obtained, within the developed system boundary, using the life cycle carbon emission (LCCE) model made through the mathematical algorithm and coded in Microsoft Excel worksheets (*see the supplementary life cycle carbon emission inventory (LCCEI) data file*). The developed LCCE data collection model was adopted to acquire the presented data [1].
Data format	*Raw and Calculated*
Parameters for data collection	The parameters for ‘activity and emission factor’ data collection are presented in excel. Those parameters are including calculated life cycle carbon emissions of the electrical power systems (LCCE,_2049_) in the year 2049, the quantity of carbon emitted in the newly added electrical power (generation and transmission) systems capacity (Qm,_2049_) in the year 2049, the carbon emission factor of the electrical product system caused by the new installation, operation, and maintenance (EFm,_2049_) in the year 2049, an electrical power system capacity (SP,_2019_) installed and survived by the base year 2019, the carbon emission factor of the electrical power system capacity installed, operated, maintained, and surviving (EFo,_2019_) in the base year 2019, the remaining quantity of carbon emissions from retired and recycled electrical product systems (RR,_2049_) in the year 2049, and remaining of the substituted recycling fraction from the electrical power systems retired (RC,_2049_) in the year 2049. The parameters related to the newly added electrical power system capacity (N,_2049_), filling quantity of a carbon emission per unit of added electrical power system capacity (A,_2049_), ratio of the carbon emission substituted into the newly added electrical power system (SA,_2049_) in the year 2049 and ratio of a carbon emission remaining in the retired electrical power system (PR,_2049_,) in the year 2049 were also studied. The data was also collected using the parameters such as the quantity of carbon emitted in the newly added electrical power (generation and transmission) systems capacity (Qm,_2048_) in the year 2048, and the retirement fraction of the newly added electrical power system (NR,_2048_) in year 2048. The established parameters have been externally peer reviewed using established questions through field survey (8 respondents), workshops, seminars, university and scientific conferences (10 respondents), and journal editors and reviewers (12 respondents).
Description of data collection	The minimum and maximum temporal data points were selected to comply with the average electrical power systems life span of 30 years from the base year 2019. The data was collected and generated for the base year 2019, the year 2048 and the year 2049. The power loss due to distribution was not included for data collection and generation just for simplification of the study. The data collection process also ignored activity and emission factor data related to the raw material acquisition (extraction and processing) and raw material transportation
	of the electrical power systems studied. The carbon emission data from biomass and bagasse energy sources was not generated since it is part of the global carbon cycle. The parameters developed from the mathematical algorithm, coded in Microsoft excel, were applied to collect existing data and developed new data. The modified mathematical representation is linked to logical relationships identified through a hypothetical LCCE approximation for the generation and transmission systems [1]. The production or reviews of activity data included information from the primary sources such as the relevant institutional actors and experts from national and international power organisations, information from secondary sources, and expert judgement. The secondary information was collected from reputable sources, such as indexed conference proceedings and journals. The collected and estimated data were re-arranged and checked for completeness, consistency, and accuracy by ensuring that the current and newly installed capacities balanced in a particular electrical power system for a targeted year. The data have also been acquired through sharing and exchange of existing, transformed, and new data through workshops and seminar presentations at the African Centre of Excellence in Energy for Sustainable Development, University of Rwanda, the postgraduate (systems) forum of the 2018 IEEE PES/IAS PowerAfrica Conference and the technical session, on the advances in energy systems, of the 2020 IEEE PES/IAS PowerAfrica Conference.
Data source location	The primary data sources were the actors of the specialised national electrical power systems institutions including: Tanzania Geothermal Company Limited (TGDC) and Tanzania Electric Supply Company (TANESCO) in Tanzania; Rwanda Energy Group (REG), Energy Utility Corporation (EUCL) and the Energy Development Corporation (EDCL) in Rwanda; and Kenya Electricity Generating Company (KenGen), Geothermal Development Company (GDC) and Kenya Electricity Transmission Company Limited (KETRACO) in Kenya. The secondary data sources used in acquiring information include: Universities’ reports, especially the additional data related to power demand in Kenya [2]; International specialised electrical power systems institutional reports, especially the additional data related to power demand in Tanzania [3]; National energy and environment reports, especially the additional data related to renewable electricity potentials in Tanzania [4]; International energy and environment reports, especially the additional data related to the hydropower potentials in Kenya [5,6]; National power systems master plans, especially the additional data related to power demand in Rwanda [7,8] International power systems master plans, especially the additional data related to the potential power sources and trade in Kenya, Rwanda and Tanzania [9]; National and international experts presentations, especially the additional activity data related to the geothermal potentials Tanzania [10]; web searches, journals and reports; Scientific and technical articles in energy and environment, especially the additional data related to the carbon emission factors for: large hydro, natural gas, geothermal [11], diesel, wind and solar [12], small hydro [13], and coal [14] potentials.
Data accessibility	Data is in this article and in a Mendeley data repository (https://data.mendeley.com/datasets/pcc8vhbvwz/2)
Related research article	E. Chambile, N. Ijumba, B. Mkandawire, and J. de D. Hakizimana, ‘Modelling of environmental emission in Kenyan, Rwandan, and Tanzanian electrical power systems’, *J. Clean. Prod.*, p. 127830, Aug. 2021.

## Value of the Data

• The acquired data can be used to evaluate the carbon intensity of the power supply systems within the established systems limits and algorithm. The obtained data can also be useful for evaluating the extent to which grid electricity generation and transmission system drivers are designed and operated in the context of environmental governance (EG) factors.
• The compiled LCCEI data can also be customised and exported to commonly used evaluation software to enhance its use for scientists, practitioners, developers and policymakers at national, regional, and global scales.
• The generated data can also enhance learnability and carbon emission monitoring of electrical power systems and sustainable sub-region and regional grid interconnections design.
• Besides it is easy to replicate the collected (existing and new) data, collected data can also openly be used by anyone who has interest in conducting a life cycle assessment of electrical power systems.
• The developed data can be openly-used for targeting and monitoring of local, regional, and global institutions for environmentally sustainable electrical power system development, and.
• The data can also be openly-used to generate additional knowledge for guiding clean technologies and the design of environmentally sustainable electricity production and transmission systems.

## 1 Data Description

The quantitative data have been collected since the obtained pilot survey data showed the developed `gate to gate LCCEI' system boundaries is acceptable for the collection of both up and downstream inventory data for electrical power systems in the study area. The installed capacity and transmission loss data has been obtained from EAPP, SAPP, REG, EDCL, EUCL, TANESCO, TGDC, KenGen, GDC and KETRACO reports and/actor(s). The technology-specific carbon emission factors have been obtained from published scientific and technical papers. The electrical power system capacity in the year 2049 has been linearly adjusted from the last year of projection presented on the most recently updated national and regional power systems master plans. The dataset was compiled after modelled life-cycle carbon emission inventory (LCCEI) parameters administration from the studied national grids for, the base year 2019 and, the projected year 2049. The dataset files in the repository provide the carbon emission factors (EFo,_2019_) of both the electrical product system capacity installed, operated, maintained, and surviving in a studied national grid for the base year 2019. The EFo, 2019 were calculated from different energy sources and presented in the dataset B1 (Cell K 8), B3. (Cell L 8) and B2 (Cell B 16). The dataset provides the carbon emission factor (EFm,_2049_) of the electrical product system caused by the new installation, operation, and maintenance in the year 2049 product presented in dataset C1 (Cell H 10), C2. (Cell G 10), and C3 (Cell J 13). The EFm,_2049_ were calculated from different energy sources and transmission loss designed for different scenarios. The power transmission system loss capacity of 0.03% has been considered. The electrical power system capacity installed and survived (SP, _2019_) values of 1565.72 MW (Tanzania), 216.23 MW (Rwanda) and 2819 MW (Kenya) have been presented for the base year 2019.

The quantity of carbon emitted in the newly added electrical power (generation and transmission) systems capacity (Qm,_2049_) in the year 2049 has been calculated in dataset D (as the product of the newly added electrical power system capacity (N,_2049_) in the year 2049, filling quantity of a carbon emission per unit of added electrical power system capacity (A,_2049_) and the ratio of the carbon emission substituted remaining into the newly added electrical power system (1-SA,_2049_) in the year 2049. The dataset D also present the carbon emissions (RR,_2049_) data remaining from the retired and recycled electrical product systems in the year 2049, as a product of the quantity of carbon emitted in the newly added electrical power (generation and transmission) systems capacity (Qm,_2048_) in the year 2048, retirement fraction of the newly added electrical power system (NR,_2048_) in the year 2048, and the ratio of a carbon emission remaining in the retired electrical power system (PR,_2049_) by the year 2049. Dataset D (Column H) presents the remaining data of the substituted recycling coefficient/the residual carbon emission fraction from the electrical power systems retired (1-RC,_2049_) in the year 2049. The higher the percentage of solar composition in the grid the lower the residual emission. The systems compose of long-lived hazardous wastes was also allocated (perceived to have) greater than 50% residue carbon emission. Dataset E presents the calculated LCCE,_2049_ (kg/MW) database of the studied grid electricity generation and transmission systems for the year 2049. Apart from the text data and Table provided by this article, the supplementary LCCEI data file (including questions, raw anonymized original responses to questions and LCCEI data (calculation toolkit/sheets) is also provided through the Mendeley data repository (https://data.mendeley.com/datasets/pcc8vhbvwz/2).

## 2 Experimental Design, Materials and Methods

**Fig. 1:**
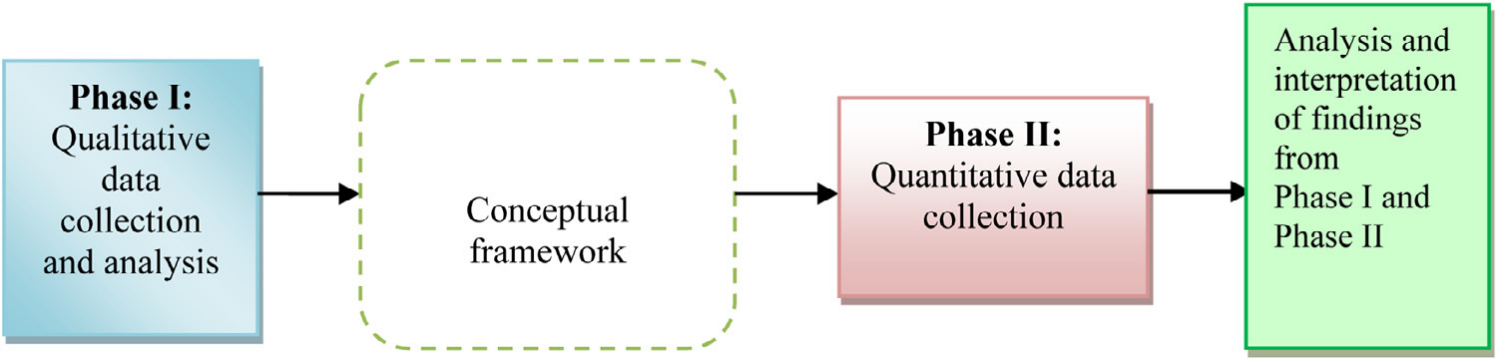
Overview of the Exploratory Sequential Design (Authors’ analysis).

The mixed methods research design was adopted whereby both qualitative and quantitative data were used to provide a better understanding of research problems as indicated in Fig. 1. This included exploratory sequential design to describe variables that are necessary for the study. The qualitative phase was used to develop the instruments and model to guide the quantitative data presented by this study. The presented data have been collected within the determined system boundary using developed LCCEI Excel worksheets of different activity and emission factor such as use of different fuel used and available energy potentials, different generation technologies, different storage technologies, different transmission technologies and established functional unit. The data acquired were documented and referenced. The life cycle carbon emission data have been converted into a common unit (kg/MW).

The possible sources of both qualitative and quantitative grid-specific life cycle carbon emission data are presented in Table 1. The grid codes, developed in Excel, have been used to collect the life cycle carbon emission data by the year 2049.

**Table 1:** Possible sources of grid-specific life cycle carbon emission data obtained from the studied countries.

National	International	Others
Specialised electrical power systems institutional actors (KenGen, KETRACO, KPLC, GDC, REG, EUCL, EDCL, TANESCO, TGDC)	Specialised electrical power systems institutional actors (Southern African Power Pool (SAPP) and Eastern Africa Power Pool (EAPP))	Scientific and technical articles in energy and environment
Energy and Environment reports	Energy and Environment reports	Journal and reports
Powers systems master plans	Powers systems master plans	Universities reports
Energy and/environmental experts, national grid systems operators	Energy and/environmental experts, sub-region grid systems operators	Web search

The preliminary survey questions planned in the content of the developed research framework and boundaries have been piloted (administered through interviews and literature review) in the sources of data identified by the researcher. The survey questions have been designed to validate the general inventory approach and assumptions. The survey has also ensured the developed research data is suitable for research question testing and avoid any potential bias. The obtained pilot survey data showed the developed `gate to gate LCCI' system boundaries is acceptable for the collection of both up and downstream inventory data for electrical power systems in the study area.

The dataset format (spreadsheets) and structure were obtained from established system limits and algorithm. The assumptions regarding power systems coverage, representative year, technology/management level were described [1]. The external peer review process and expert opinion regarding the developed research questions, system boundary, mathematical algorithm, and its underlying data were adopted to ensure database validity and utility. The use of expert judgment has been applied to determine the appropriate way to apply a model, appropriate mix of technologies, appropriate activity and emission factor data, and appropriate regression techniques to reduce possible bias and increase accuracy.

The use of national data has been preferred since sources are typically more up to date and provide better links to the originators of data. In some cases, directly applicable data was not available, therefore physically and statistically related alternative data that have a correlation with the missing data were applied to develop the LCCEI spreadsheet database. The Excel worksheets B1, B2, B3, C1, C2 and C3, of the LCCEI data file presented in a Mendeley data repository linked to this article, were applied to collect and/calculate grid electricity generations/consumptions data (for each fuel type for each end-use), transmission systems loss data (for each studied grid), and their respective life cycle carbon emission factor data. Excel worksheets D, of the presented LCCEI data file, has been used to calculate LCCE,_2049_ of a particular year using the parametric values (N,_2049_, A,_2049_, NR,_2049_, SA, _2049_, RC,_2049_, RR,_2049_, SP,_2019_, Qm,_2049_, Qm,_2048_, EFo,_2019_, and EFm,_2049_) estimated based on the established mathematical algorithm, system boundary model, scenarios, and the key assumptions. The LCCE data have been computed from the available and assumed data using Eq. (1) slightly modified from the previously published research article [1], and expressed as follows:

(1) LCCE,2049=Qm,2049×EFm,2049+SP,2019×EFo,2019+RR,2049(1−RC,2049)

Where:

• LCCE,_2049_ calculated life cycle electrical power systems amount of carbon emission (kg/MW) in the year 2049,
• Qm,_2049_ represents the quantity of carbon emitted in the newly added electrical power (generation and transmission) systems capacity (kg/MW) in the year 2049,
• EFm,_2049_ represents a carbon emission factor (kg/MW) of the electrical product system caused by the new installation, operation, and maintenance in the year 2049,
• SP, _2019_ is an electrical power system capacity (MW) installed and survived by the base year 2019,
• EFo,_2019_ represents a carbon emission factor (kg/MW) of the electrical power system capacity installed, operated, maintained, and survived by the base year 2019,
• RR,_2049_ represents the remaining quantity of carbon emissions (kg/MW) from retired and recycled electrical product systems in the year 2049, and
• RC,_2049_ represents a remaining of the substituted recycling coefficient/the residual carbon emission fraction from the electrical power systems retired in the year 2049.

The Qm,_2049_ data have been computed from the available and assumed data using Eq. (2) slightly modified from the previously published research article [1], and expressed as follows:

(2) Qm,2049=N,2049×A,2049(1−SA,2049)

Where:

• N,_2049_ represents the newly added electrical power system capacity (MW) in the year 2049,
• A_2049_ represents the filling quantity of a carbon emission per unit of added electrical power system capacity (kg/MW) in the year 2049, and
• SA,_2049_ represents the ratio of the carbon emission substituted into the newly added electrical power system in the year 2049.

The RR,_2049_ data have also been computed from the available and assumed data using Eq. (3) slightly modified from the previously published research article [1], and expressed as follows:

(3) $$\mathrm{RR},2049=\Sigma_{j=1}^{n}\mathrm{Qm},2048\times \mathrm{NR},2048\times \mathrm{PR},2049$$

Where:

• PR,_2049_ represents the ratio of a carbon emission remaining in the retired electrical power system,
• Qm,_2048_ represents the quantity of carbon emitted in the newly added electrical power (generation and transmission) systems capacity (kg/MW) in the year 2048, and
• NR,_2048_ is the retirement fraction of the newly added electrical power system in the year 2048.

## Ethics Statement

The respective ethical operation guidelines, and procedures, were considered during the research and publication process [1]. This data section reports raw and calculated data recorded at the developed LCCE inventory model. No data collected from social media platforms were presented. The contribution of all authors is well mentioned. Ethics approval for survey studies is not mandatory, since this work does not involve the use of human subjects or animal experiments. However, the ethics of this study was approved by the doctoral committee, of the African Centre of Excellence in Energy for Sustainable Development at the College of Science and Technology, of the University of Rwanda, in Rwanda and also approved by the Sokoine University of Agriculture in Tanzania. Permission to the companies and actors directly involved in the study has also been granted. Where necessary, participants or participant data were fully anonymized while complying with all data redistribution policies from the platform(s).

## Funding Source

This work was supported by the African Centre of Excellence in Energy for Sustainable Development through the World Bank's African Centre of Excellence II Project.

## CRediT authorship contribution statement

**Enock Chambile:** Conceptualization, Methodology, Investigation, Formal analysis, Data curation, Funding acquisition, Writing – original draft. **Nelson Ijumba:** Supervision, Writing – review & editing, Validation. **Burnet Mkandawire:** Supervision, Writing – review & editing, Validation. **Jean de Dieu Hakizimana:** Supervision, Validation, Project administration.

## Declaration of Competing Interest

The authors declare that they have no known competing financial interests or personal relationships that could have appeared to influence the work reported in this paper.
